# Supplementary material for: Nucleolar Stress Functions Upstream to Stimulate Expression of Autophagy Regulators
Source: Cancers (Basel). 2021 Dec 10;13(24):6220. doi: 10.3390/cancers13246220 (PMC8699128; doi:10.3390/cancers13246220)
Supplement: Supplementary file 1 [file cancers-13-06220-s001.zip › cancers-1486660-supplementary.pdf]

cancers

Article

Supplementary Materials: Nucleolar Stress Functions Upstream to Stimulate Expression of Autophagy Regulators

David P. Dannheisig, Anna Schimansky, Cornelia Donow and Astrid S. Pfister *

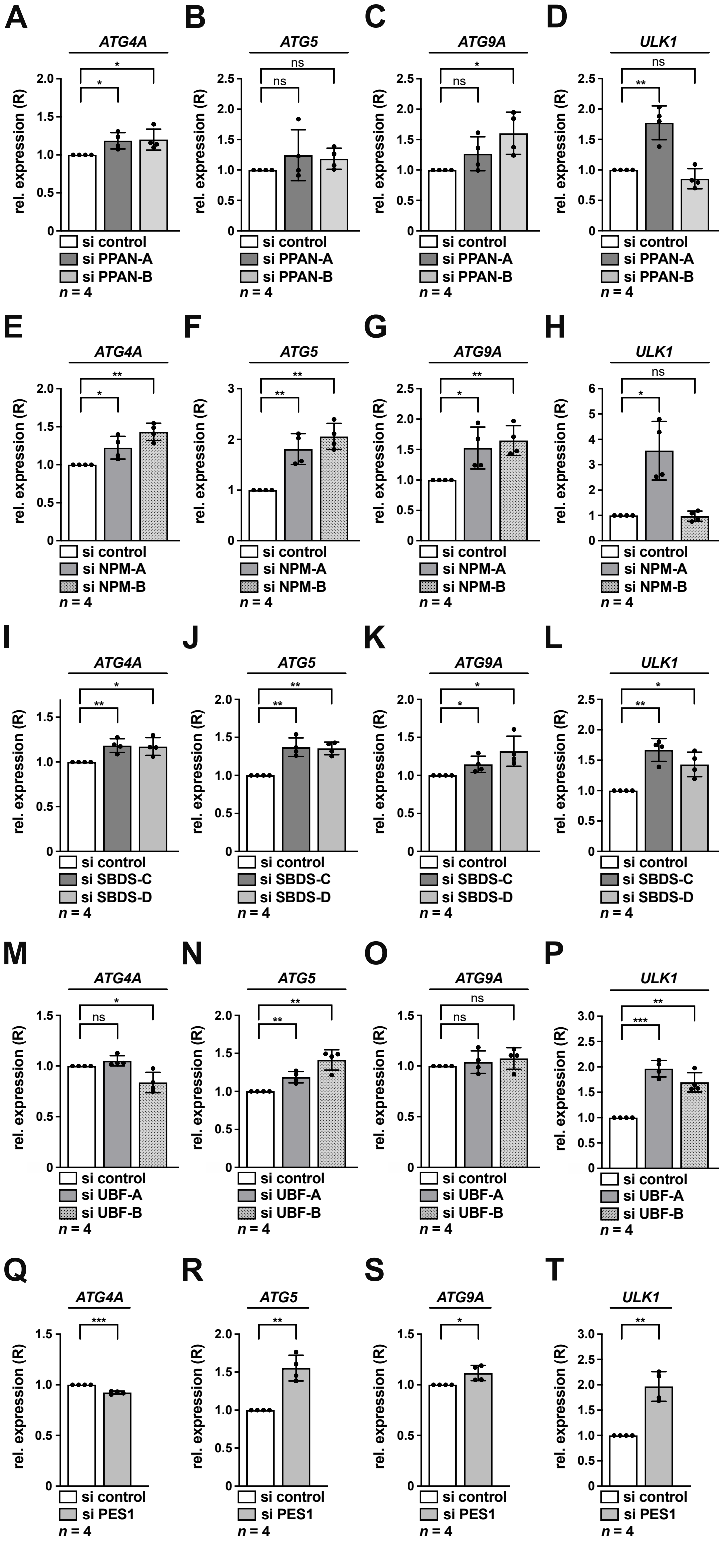

Figure S1. Impact of PPAN, NPM, SBDS, UBF-1 or PES1 depletion on mRNA levels of *ATG4A*, *ATG5*, *ATG9A* and *ULK1* in HeLa cells. HeLa cells were transfected with PPAN **(A–D)**, NPM **(E-H)**, SBDS **(I–L)**, UBF **(M–P)** or PES1 **(Q–T)** si RNAs for 48 h as indicated and subsequently analyzed by qRT-PCR. Of note, the respective samples correspond to Figure 2 and Figure 3. Relative expression (R) of *ATG4A* **(A,E,I,M,Q)**, *ATG5* **(B,F,J,N,R)**, *ATG9A* **(C,G,K,O,S)** or *ULK1* **(D,H,L,P,T)** normalized to *GAPDH* is shown. The si control was set to 1. Error bars represent S.D., *p* values were calculated by t-test. *, $p < 0.05$, **, $p < 0.01$, ***, $p < 0.001$, ns, non-significant. *n* = number of independent experiments.

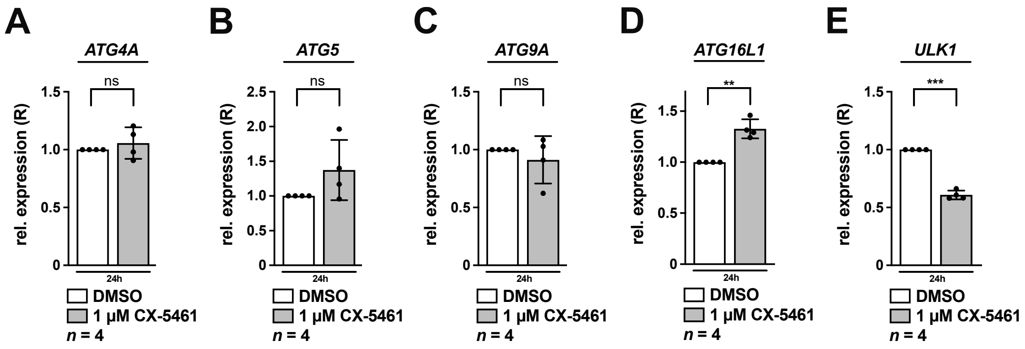

Figure S2. Influence of CX-5461 treatment on mRNA levels of *ATG4A*, *ATG5*, *ATG9A*, *ATG16L1* and *ULK1* in HeLa cells. HeLa cells were incubated with 1 µM CX-5461 for 24 h and subsequently analyzed by qRT-PCR. Of note, the respective samples correspond to Figure 6. Relative expression (R) of *ATG4A* **(A)**, *ATG5* **(B)**, *ATG9A* **(C)**, *ATG16L1* **(D)** or *ULK1* **(E)** normalized to *GAPDH* is shown. The si control was set to 1. Error bars represent S.D., *p* values were calculated by t-test. **, $p < 0.01$, ***, $p < 0.001$, ns, non-significant. *n* = number of independent experiments.

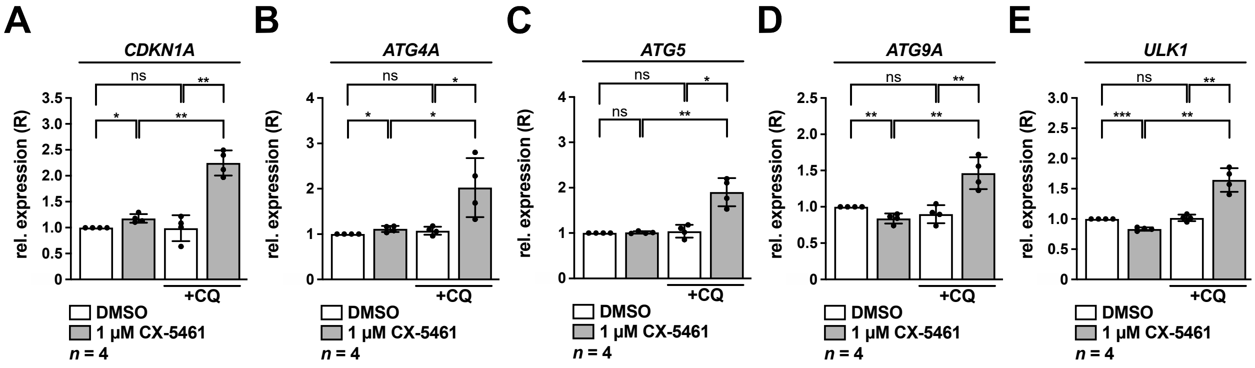

Figure S3. Inhibition of RNA pol I by CX-5461 results in increased expression of *CDKN1A*, *ATG4A*, *ATG5*, *ATG9A* and *ULK1* upon CQ treatment. HEK293A GFP-LC3 cells were incubated with 1 µM CX-5461 for 20.5 h and subsequently exposed to chloroquine (CQ) for 3.5 h as indicated prior qRT-PCR analysis. Of note, the respective samples correspond to Figure 7. Relative expression (R) of *CDKN1A* **(A)**, *ATG4* **(B)**, *ATG5* **(C)**, *ATG9A* **(D)** or *ULK1* **(E)** normalized to *GAPDH* is shown. The si control was set to 1. Error bars represent S.D., *p* values were calculated by t-test. *, $p < 0.05$, **, $p < 0.01$, ***, $p < 0.001$, ns, non-significant. *n* = number of independent experiments

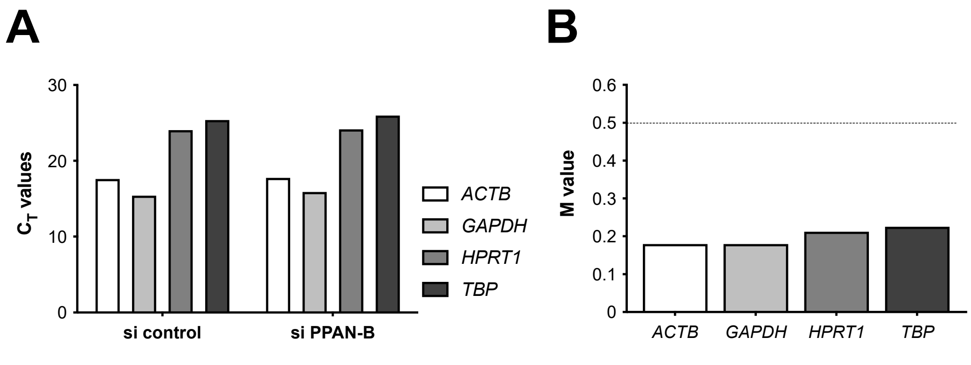

Figure S4. Stability of reference genes investigated in the background of PPAN knockdown qRT-PCR experiments. HeLa cells were transfected with si control or si PPAN-B for 48 h and subsequently analyzed by qRT-PCR via subjection to a predesigned PrimePCR™ Autophagy panel (BioRad) whereas the respective threshold cycles (C_T) of *ACTB*, *GAPDH*, *HPRT1* or *TBP* are shown in **(A)** and the M value for each reference gene in **(B)**. Of note, the utilized samples correspond to Figure 1.

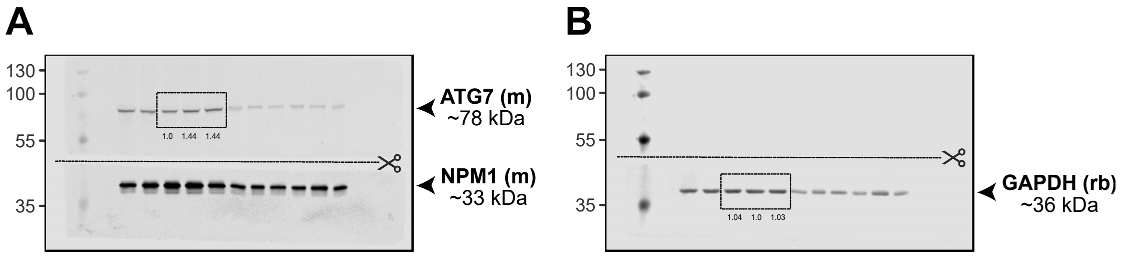

Figure S5. Raw Western blot data corresponding to Figure 2D. Densitometric result of the scanned 800 nm **(A)** and 680 nm **(B)** channel. The rectangle represents the clipping, which is depicted in the main manuscript. Below, the respective fold-change is indicated. The horizontal line and scissor indicate where the blot was cut. The protein marker is detected in the 680 nm channel, the molecular weight (kDa) is depicted on the left. The utilized primary antibodies are indicated on the right with additional information on the host species (m: mouse, rb: rabbit) and the calculated molecular weight.

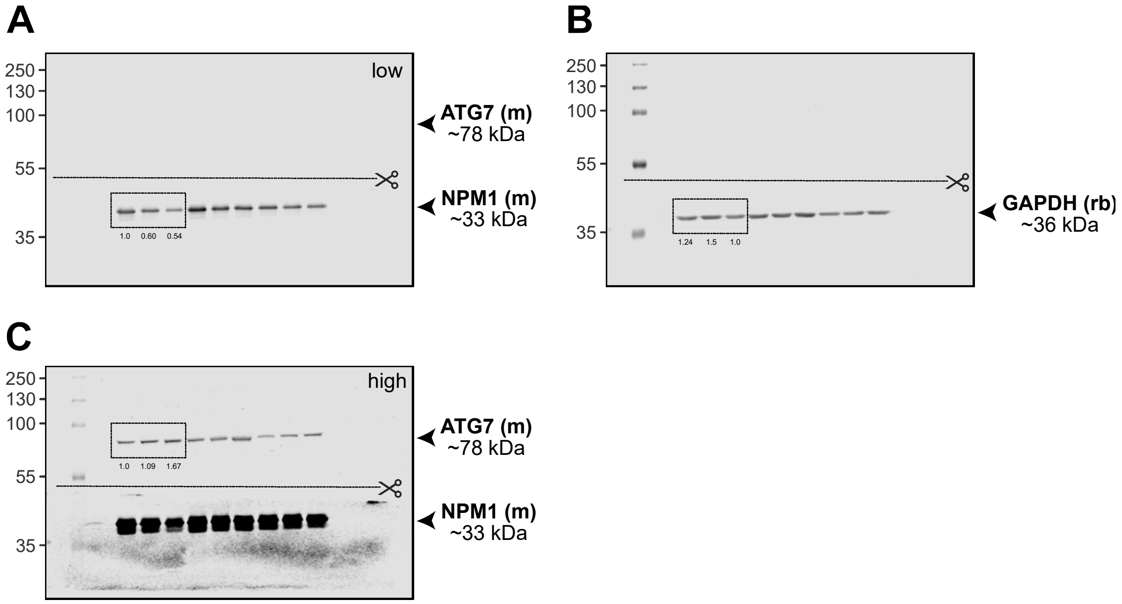

Figure S6. Raw Western blot data corresponding to Figure 5A. Densitometric result of the scanned 800 nm **(A,C)** and 680 nm **(B)** channel. The rectangle represents the clipping, which is depicted in the main manuscript. Below, the respective fold-change is indicated. The horizontal line and scissor indicate where the blot was cut. The protein marker is detected in the 680 nm channel, the molecular weight (kDa) is depicted on the left. The utilized primary antibodies are indicated on the right with additional information on the host species (m: mouse, rb: rabbit) and the calculated molecular weight. Low = low exposure, high =high exposure.

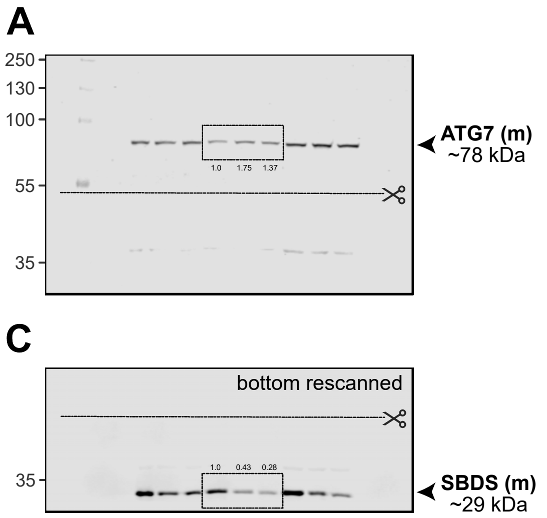

Figure S7. Raw Western blot data corresponding to Figure 5D. Densitometric result of the scanned 800 nm **(A,C)** and 680 nm **(B)** channel. The rectangle represents the clipping, which is depicted in the main manuscript. Below or above, the respective fold-change is indicated. The horizontal line and scissor indicate where the blot was cut. The protein marker is detected in the 680 nm channel, the molecular weight (kDa) is depicted on the left. The utilized primary antibodies are indicated on the right with additional information on the host species (m: mouse, rb: rabbit) and the calculated molecular weight.

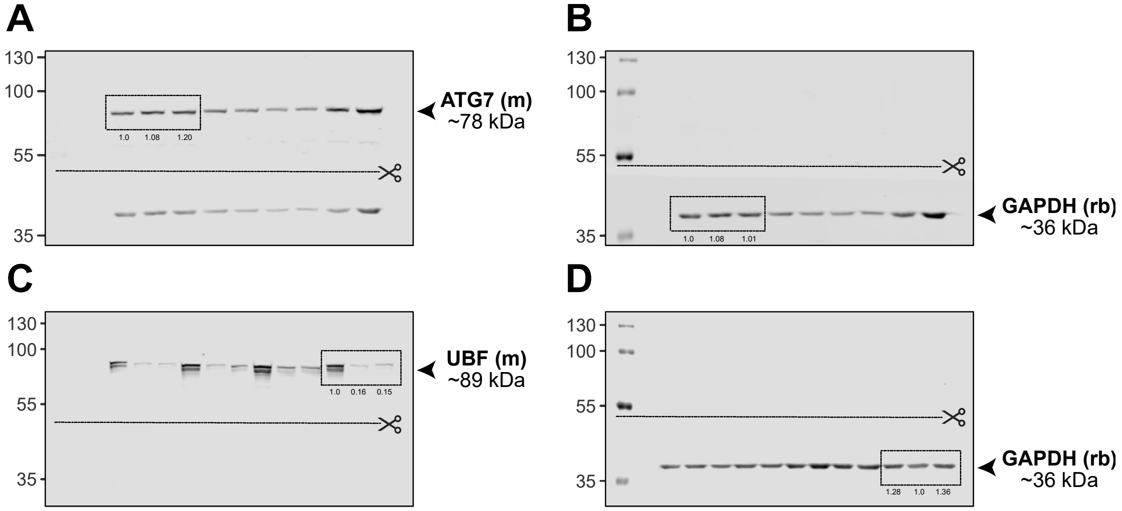

Figure S8. Raw Western blot data corresponding to Figure 5G. Densitometric result of the scanned 800 nm **(A,C)** and 680 nm **(B,D)** channel. (**A**) and (**B**) or (**C**) and (**D**) corresponds to the same blot. The rectangle represents the clipping, which is depicted in the main manuscript. Below the respective fold-change is indicated. The horizontal line and scissor indicate where the blot was cut. The protein marker is detected in the 680 nm channel, the molecular weight (kDa) is depicted on the left. The utilized primary antibodies are indicated on the right with additional information on the host species (m: mouse, rb: rabbit) and the calculated molecular weight.

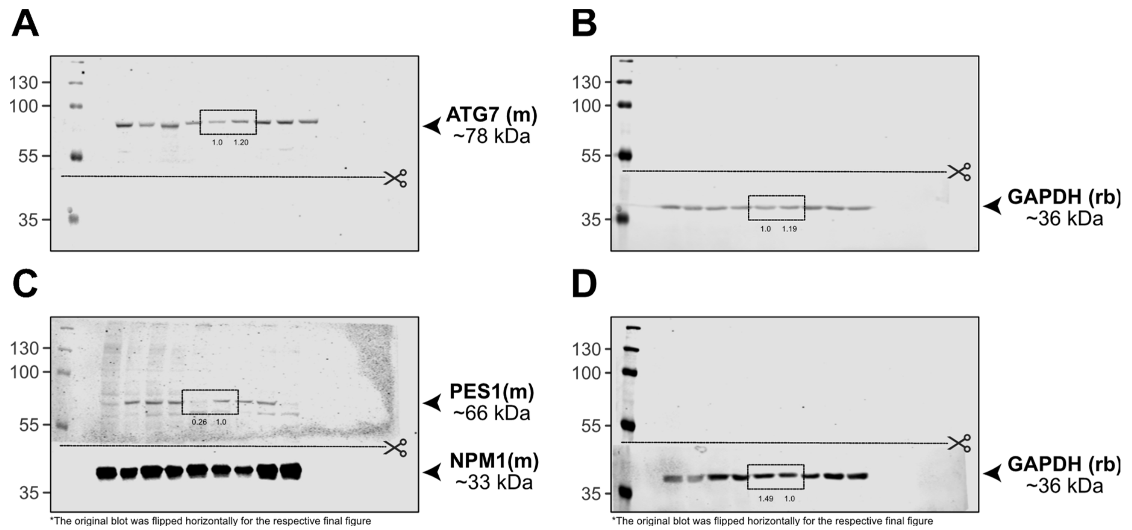

Figure S9. Raw Western blot data corresponding to Figure 5J. Densitometric result of the scanned 800 nm **(A,C)** and 680 nm **(B,D)** channel. (**A**) and (**B**) or (**C**) and (**D**) corresponds to the same blot. The rectangle represents the clipping, which is depicted in the main manuscript. Below the respective fold-change is indicated. The horizontal line and scissor indicate where the blot was cut. The protein marker is detected in the 680 nm channel, the molecular weight (kDa) is depicted on the left. The utilized primary antibodies are indicated on the right with additional information on the host species (m: mouse, rb: rabbit) and the calculated molecular weight.

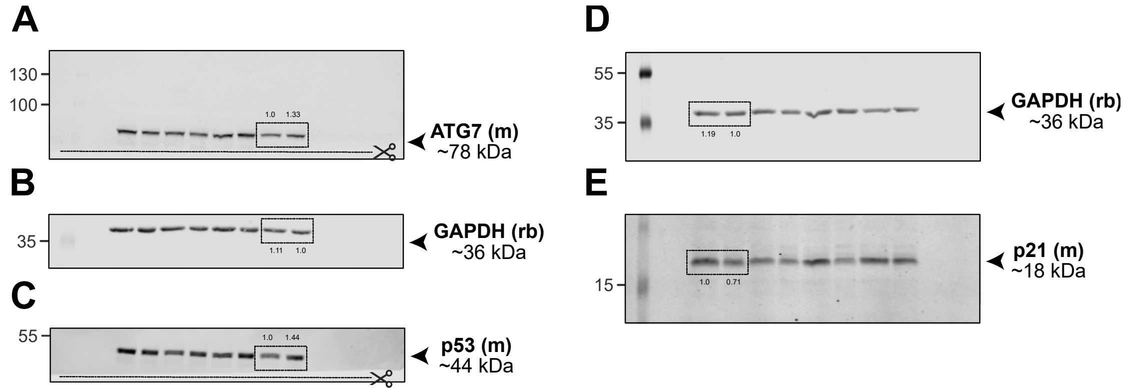

Figure S10. Raw Western blot data corresponding to Figure 6E. Densitometric result of the scanned 800 nm **(A,C,E)** and 680 nm **(B,D)** channel. (**A–C**) or (**D**) and (**E**) corresponds to the same blot. The rectangle represents the clipping, which is depicted in the main manuscript. Below or above, the respective fold-change is indicated. The horizontal line and scissor indicate where the blot was cut. The protein marker is detected in the 680 nm channel, the molecular weight (kDa) is depicted on the left. The utilized primary antibodies are indicated on the right with additional information on the host species (m: mouse, rb: rabbit) and the calculated molecular weight.

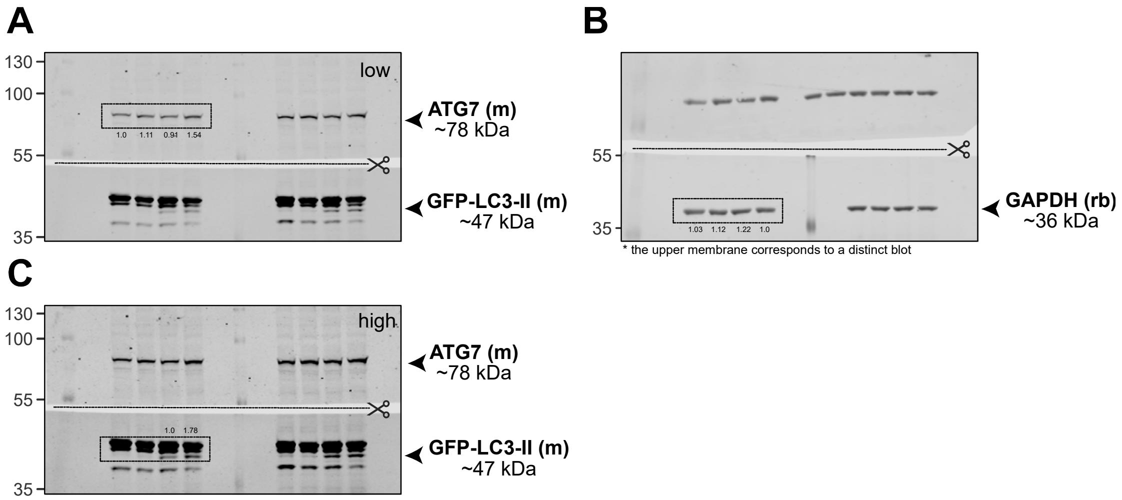

Figure S11. Raw Western blot data corresponding to Figure 7D. Densitometric result of the scanned 800 nm **(A,C)** and 680 nm **(B)** channel. The rectangle represents the clipping, which is depicted in the main manuscript. Below the respective fold-change is indicated. The horizontal line and scissor indicate where the blot was cut. The protein marker is detected in the 680 nm channel, the molecular weight (kDa) is depicted on the left. The utilized primary antibodies are indicated on the right with additional information on the host species (m: mouse, rb: rabbit) and the calculated molecular weight. Low = low exposure, high =high exposure.
